# Programmed Death-1 Inhibitor Immunotherapy-Induced Symptomatic Toxic Thyroid Nodule

**DOI:** 10.7759/cureus.54143

**Published:** 2024-02-13

**Authors:** Owen Cole, Prahasith Kamani, Vikash Kumar, Jacob Warman

**Affiliations:** 1 Internal Medicine, The Brooklyn Hospital Center, Brooklyn, USA; 2 Endocrinology, The Brooklyn Hospital Center, Brooklyn, USA

**Keywords:** programmed death ligand 1 inhibitor, hot nodule, thyrotoxicosis, merkel cell carcinoma, hyperthyroidism

## Abstract

Immune checkpoint inhibitors, specifically programmed death-ligand 1 (PD-LI) inhibitors, are immune modifying medications that increasingly treat specific types of cancer. They are known to cause many side effects, including thyroid-related side effects. The use of PD-L1 inhibitors can cause hypothyroidism most commonly, while hyperthyroidism occurs less frequently. This case report describes a patient who developed a toxic thyroid nodule while taking the PD-L1 inhibitor, avelumab, for the treatment of Merkel cell carcinoma. It highlights the need for more research into the specific mechanisms by which these therapies can cause hyperthyroidism. It also raises the question regarding the association between the use of these medications and the development or worsening of thyroid nodules.

## Introduction

Immune checkpoint inhibitors (ICI) are targeted therapies that assist immune-mediated destruction of tumor cells. Programmed death-ligand 1 (PD-L1) inhibitors are a class of ICI that target the PDL1 pathway, which regulates the immune response and prevents excessive immune activity. Certain cancers such as melanoma, lung carcinoma, head and neck squamous cell carcinoma, urothelial carcinoma, and multiple hematological malignancies exploit this pathway to evade immune surveillance. Programmed death receptor 1 (PD-1) is found in various immune cells such as T cells and B cells. Tumor cells can alter this PD-1/PD-L1 interaction to induce and maintain immune tolerance. Activation of the PD-1/PD-L1 pathway inhibits T-cell activation, promotes apoptosis of tumor-infiltrating lymphocytes, and decreases secretion of inflammatory cytokines, eventually resulting in the escape of tumor cells from immune surveillance. PD-L1 inhibitors are monoclonal antibodies targeting PD-L1 on the surface of the tumor preventing the hijacking of the PD-1/PD-L1 axis to evade destruction [1,2].

In recent years, ICI has emerged as a cornerstone treatment modality across various cancer types, significantly reshaping the therapeutic landscape in cancer management. Due to their specific targets and mechanisms of action, ICI can induce immune-related adverse events, leading to autoimmune and inflammatory effects. These predominantly manifest in the skin, gastrointestinal tract, liver, and endocrine system.

This case report highlights a unique presentation of hyperthyroidism associated with a hot thyroid nodule in a patient taking a PDL1 inhibitor, specifically avelumab, for the treatment of Merkel cell carcinoma. It is notable as there is little to no existing literature documenting the induction or exacerbation of toxic multinodular goiters or toxic autonomous nodules associated with immune checkpoint inhibitor therapy. Our case report presents a unique association between a hot-functioning thyroid nodule and the use of a PD-L1 inhibitor [1,2].

## Case presentation

**Figure 1:**
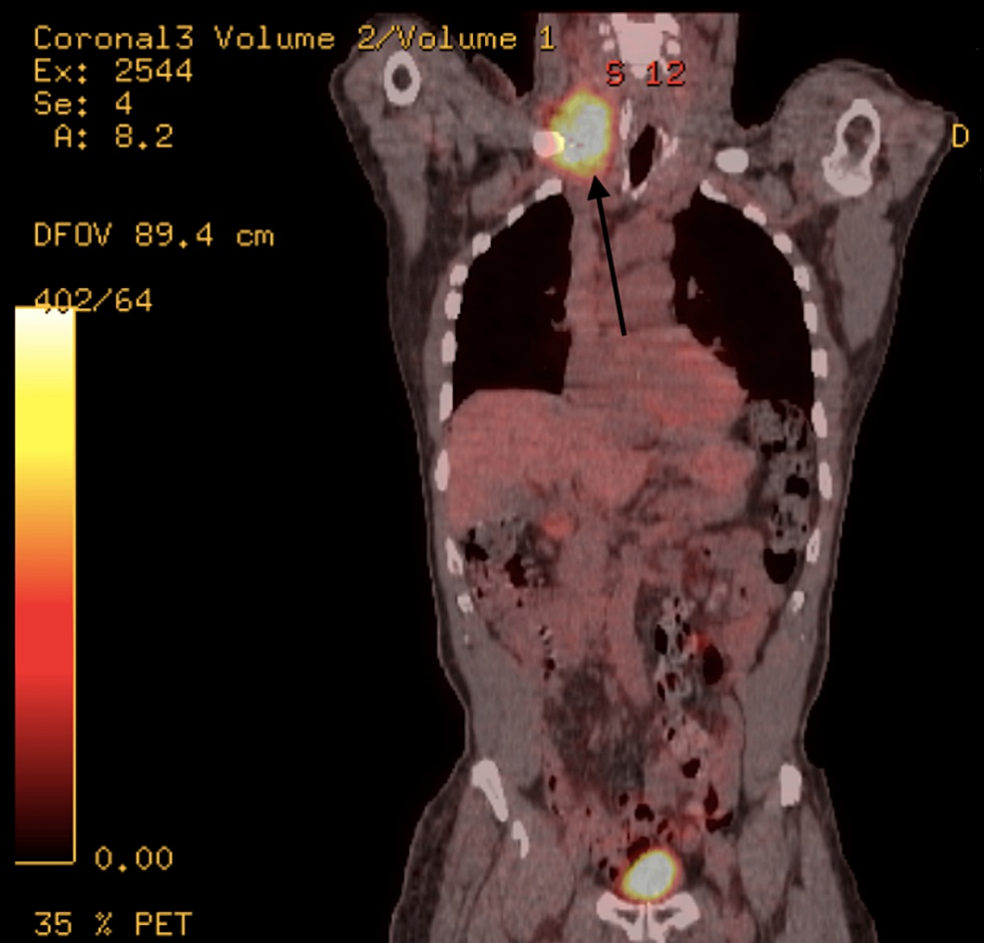
PET scan showing the hypermetabolic region in the right neck consistent with matted adenopathy (arrow) PET: Positron emission tomography.

**Figure 2:**
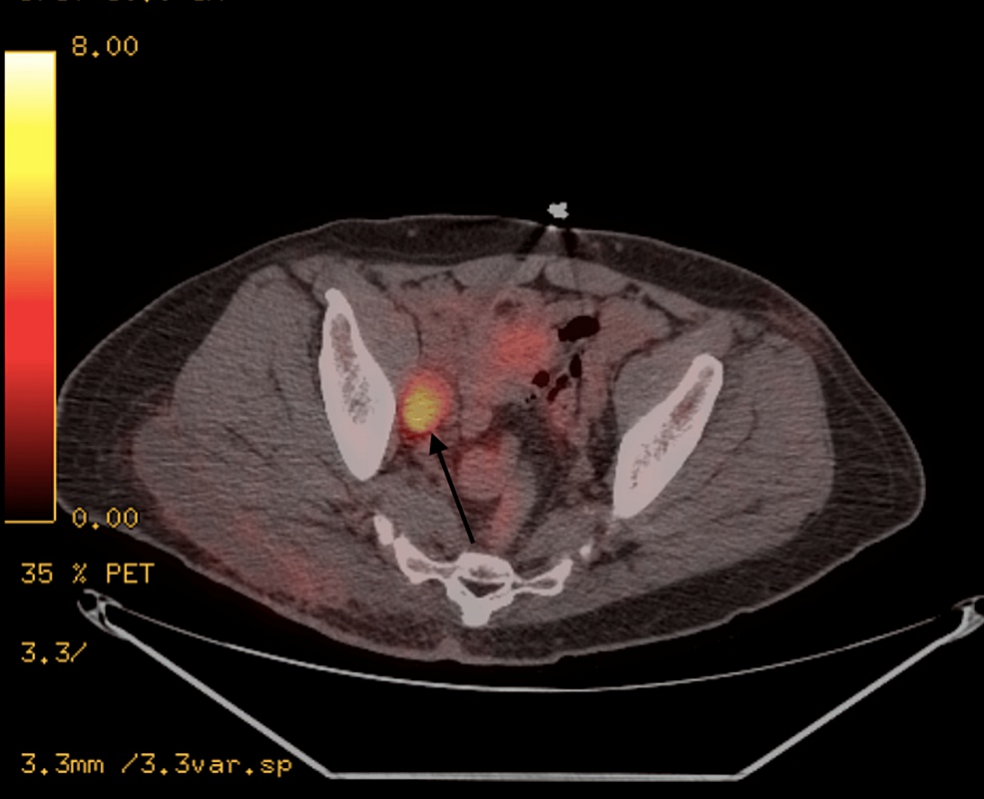
PET scan showing the hypermetabolic right obturator lymph node (arrow) PET: Positron emission tomography.

An 82-year-old male with a history of hypertension presented to the endocrinology department with complaints of weight loss and the presence of a thyroid nodule. The patient had previously been diagnosed with stage IIIB Merkel cell carcinoma of the skin, which was confirmed through histopathology following an excisional biopsy of a large cutaneous lesion on the right buttock. The initial biopsy revealed a 6.5 x 5.5 x 4 cm Merkel cell carcinoma with evidence of vascular invasion and positive margins. Subsequently, wide excision was performed, and a positron emission tomography-computed tomography (PET-CT) scan was conducted for staging purposes. The scan indicated no residual disease in the right buttock, but it identified a hypermetabolic right obturator lymph node, hypermetabolic right inguinal lymph node, and hypermetabolic region in the right neck consistent with matted adenopathy, which raised suspicion of metastatic lymph node involvement (Figures 1, 2).

Based on these findings, the patient was staged as III B (T3, N1b, Pm0). A biopsy of the right inguinal lymph node confirmed metastatic disease. The patient was referred to radiation oncology and received external beam radiation therapy. Six months after the initial diagnosis, a follow-up PET-CT scan revealed the presence of multiple new hypermetabolic lymph nodes on the right side of the neck, at the gastroesophageal junction, and a new subcutaneous nodule on the left buttock, indicative of metastatic disease. As a result, the patient began treatment with avelumab, a PD-L1 inhibitor. Throughout the immunotherapy treatment, the patient's thyroid-stimulating hormone (TSH) and adrenocorticotropic hormone (ACTH) levels were monitored for any associated endocrinopathies (Table 1). Baseline thyroid function tests were obtained before initiating treatment; however, an ultrasound of the thyroid was not obtained.

**Table 1:** Thyroid function tests TSH: Thyroid-stimulating hormone.

	Before avelumab therapy	Before methimazole initiation	After initiation of methimazole
TSH (0.35-4.94 mciu/ml)	3.71	0.00	2.30
Free T4 (0.7-1.5 ng/dl)	1.2	2.4	1.1
Total T3 (0.35-1.93 ng/dl)	0.6	2.1	1.3

Two months after initiating immunotherapy, after receiving four cycles of avelumab, the patient reported weight loss (15 pounds) and a palpable lump on his neck. He also complained of anxiety, heat intolerance with increased diaphoresis, and intermittent palpitations. His oncologist referred him to the endocrine clinic for evaluation of a suspected thyroid nodule. A physical exam at that time showed a thin, elderly male with a regular cardiac and pulmonary exam, who was significant for a palpable nodule on the left side of the thyroid. A repeat TSH level showed suppression (TSH level of zero) with elevated levels of T4 and T3, a change from a normal thyroid profile four months prior (Table 1).

**Figure 3:**
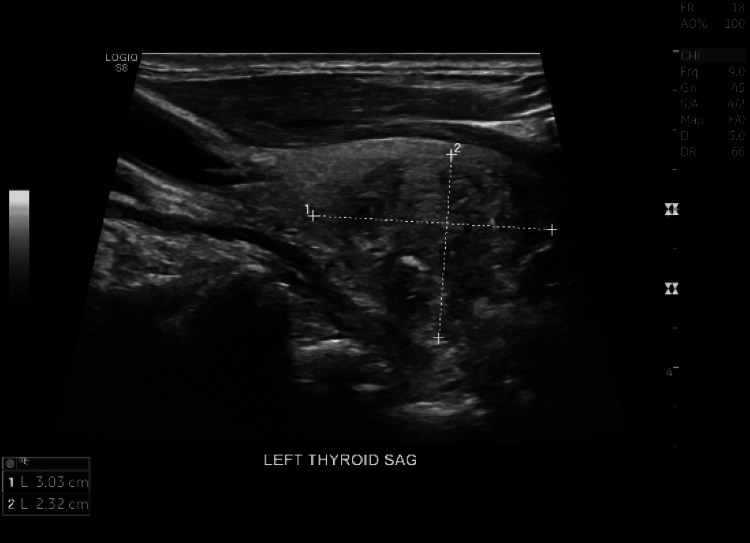
Ultrasound thyroid showing the mass occupying the left mid to lower pole, measuring 3.0 x 1.9 x 2.3 cm

To rule out autoimmune causes of thyrotoxicosis, antibody levels were measured. Thyroid-stimulating immunoglobulin, TSH antibody, thyroid peroxidase antibody, and thyroglobulin antibody were all negative. There were no prior thyroid ultrasounds to compare with, but an ultrasound of the thyroid revealed a mass occupying the left mid to lower pole, measuring 3.0 x 1.9 x 2.3 cm (Figure 3).

**Figure 4:**
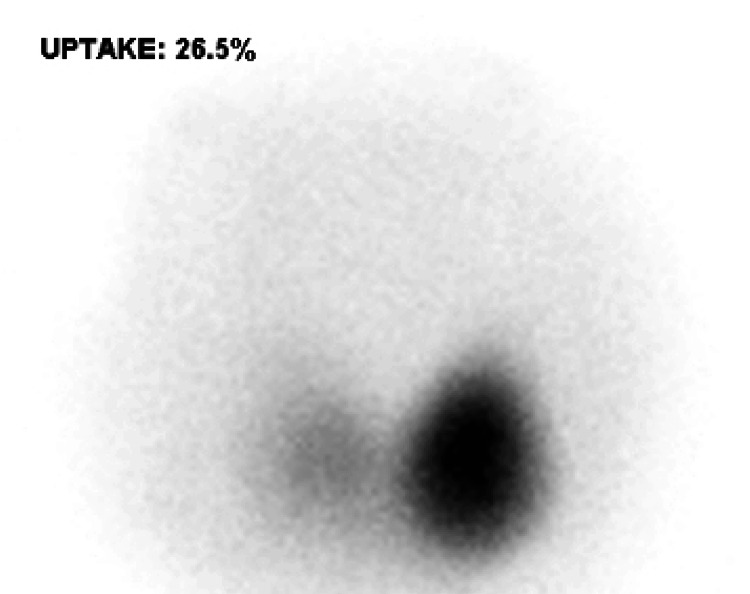
Thyroid uptake scan showing large hot nodule (3 x 2.5 cm) in the left mid and lower poles, causing suppression of the remaining thyroid gland lobes

A nuclear medicine scan of the thyroid demonstrated high normal thyroid uptake with a large hot nodule (3 x 2.5 cm) in the left mid and lower poles, causing suppression of the remaining thyroid gland lobes, suggestive of an autonomous hot nodule (Figure 4). The patient was subsequently initiated on methimazole treatment, and avelumab was withheld. Clinically, the patient demonstrated improvement, and follow-up thyroid profile tests showed subsequent improvement as well (Table 1).

**Figure 5:**
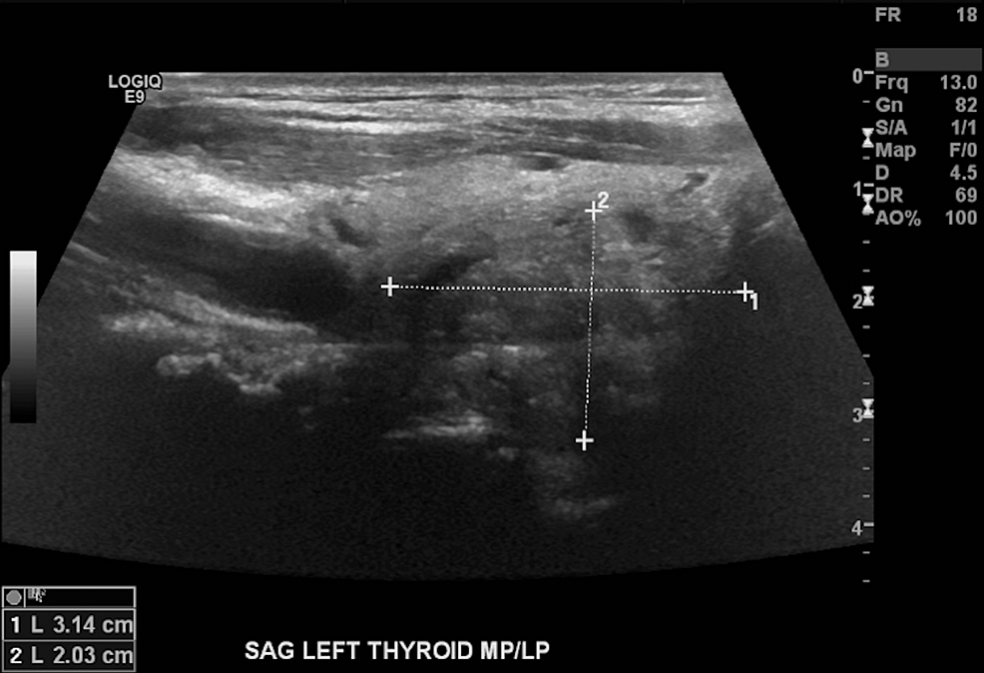
Thyroid ultrasound after treatment with methimazole showing a nodule in the mid to lower pole on the left, measuring 3.1 x 2 x 2 cm

After normalization of thyroid levels and symptomatic improvement and at the discretion of his oncologist, the patient was then started on ipilimumab/nivolumab combination therapy. A repeat thyroid ultrasound was obtained six months after starting methimazole while on ipilimumab/nivolumab, which showed a persistent thyroid nodule despite improvement in thyroid function tests and symptoms (Figure 5). This suggests that the patient likely developed a toxic hot nodule after initiating avelumab therapy, which persisted despite discontinuing the immunotherapy. He continued regular follow-up with endocrinology, and his thyroid function remained stable.

## Discussion

Thyroid anomalies such as hypothyroidism, thyroiditis, and hyperthyroidism are extremely common. The precise mechanism underlying the development of hyperthyroidism in PD-L1 inhibitors is not fully understood. However, it is believed to involve immune-mediated mechanisms resulting in the activation of T cells and the production of autoantibodies that stimulate the thyroid gland. One proposed mechanism is that PD-L1 inhibitors enhance the activation of T cells, which can lead to increased T-cell infiltration into the thyroid gland. This infiltration triggers an immune response against thyroid antigens, causing inflammation and subsequent thyroid dysfunction. Another possible mechanism involves the disruption of the immune tolerance of regulatory T cells (Tregs). PD-L1 inhibitors can interfere with the PD-1/PD-L1 pathway, which plays a role in maintaining immune self-tolerance. By blocking this pathway, the balance between effector T cells and Tregs may be disrupted, leading to an excessive immune response against the thyroid gland [1,2].

ICI-related thyroid dysfunction typically manifests around six weeks after initiating ICI therapy, although it can occur at any point during treatment. Patients who develop ICI-related hyperthyroidism can be asymptomatic and detected on routine lab monitoring or may present with symptoms such as weight loss, anxiety, palpitations, and tremors. Severe thyrotoxicosis such as thyroid storm is quite rare but has been reported in case reports. Development of post-ICI Grave’s disease has also been described in a few case reports [3].

All patients treated with ICIs should have their thyroid function assessed at baseline and every four to eight weeks or more frequently if clinically indicated. When a low TSH is found with elevated free T4 and/or T3, a diagnosis of thyrotoxicosis is suggested. However, if both low TSH and low free T4 are found, then central hypothyroidism should be diagnosed, and patients should be considered for thyroid hormone replacement accordingly. The evaluation of ICI-induced hyperthyroidism typically involves conducting a thyroid ultrasound (USG) and performing radioactive iodine uptake (RAIU) scans to assess the underlying etiology [1,4]. While ICIs have been associated with both hyperthyroidism and hypothyroidism, there is relatively little research into their association with the developing or worsening of thyroid nodules. It is important to note that the development of hyperthyroidism in PD-L1 inhibitors is relatively less common compared to hypothyroidism, and further research is needed to fully elucidate the underlying mechanisms.

## Conclusions

Thyroid dysfunction is a common side effect to be considered in patients treated with ICI such as PD-1/PD-L1 inhibitors. While hypothyroidism is more commonly seen in patients treated with PD-L1 inhibitors, our case report shows a patient presenting with thyrotoxicosis and hyperthyroidism with the development of a hot-functioning thyroid nodule, post the avelumab (PD-L1 inhibitor) therapy. This highlights a unique thyroid complication of PD-L1 therapy. Overall, this case underlines the importance of monitoring patients for endocrinological dysfunction post immune-modulating therapies such as PD-1/PD-L1 inhibitors and the need for further research on endocrinopathies associated with these ICIs to understand the underlying mechanisms in greater detail. It also highlights the need for more research into the relationship between ICI and the development or worsening of thyroid nodules. It also highlights the need for specific guidelines for the monitoring of thyroid function while on PD-1/PDL-1 inhibitors.
